# Proliferation of Purple Sulphur Bacteria at the Sediment Surface Affects Intertidal Mat Diversity and Functionality

**DOI:** 10.1371/journal.pone.0082329

**Published:** 2013-12-05

**Authors:** Cédric Hubas, Bruno Jesus, Mickael Ruivo, Tarik Meziane, Najet Thiney, Dominique Davoult, Nicolas Spilmont, David M. Paterson, Christian Jeanthon

**Affiliations:** 1 Muséum National d’Histoire Naturelle, UMR BOREA 7208 MNHN/CNRS/IRD/UPMC, Paris, France; 2 LUNAM université, Université de Nantes, Mer Molécules Santé EA 2160, Faculté des Sciences et des Techniques, Nantes, France; 3 Centro de Biodiversidade, Genómica Integrativa e Funcional (BioFIG), Faculdade de Ciências, Universidade de Lisboa, Lisboa, Portugal; 4 UPMC Univ Paris 06, UMR 7144, Adaptation et Diversité en Milieu Marin, Station Biologique de Roscoff, Roscoff, France; 5 CNRS, UMR 7144, Adaptation et Diversité en Milieu Marin, Station Biologique de Roscoff, Roscoff, France; 6 CNRS, UMR 8187, LOG, Station Marine de Wimereux, Wimereux, France; 7 Sediment Ecology Research Group, Scottish Ocean Institute, University of St Andrews, St Andrews, Scotland, United Kingdom; CSIR- National institute of oceanography, India

## Abstract

There is a relative absence of studies dealing with mats of purple sulphur bacteria in the intertidal zone. These bacteria display an array of metabolic pathways that allow them to disperse and develop under a wide variety of conditions, making these mats important in terms of ecosystem processes and functions. Mass blooms of purple sulphur bacteria develop during summer on sediments in the intertidal zone especially on macroalgal deposits. The microbial composition of different types of mats differentially affected by the development of purple sulphur bacteria was examined, at low tide, using a set of biochemical markers (fatty acids, pigments) and composition was assessed against their influence on ecosystem functions (sediment cohesiveness, CO_2_ fixation). We demonstrated that proliferation of purple sulphur bacteria has a major impact on intertidal mats diversity and functions. Indeed, assemblages dominated by purple sulphur bacteria (Chromatiaceae) were efficient exopolymer producers and their biostabilisation potential was significant. In addition, the massive growth of purple sulphur bacteria resulted in a net CO_2_ degassing whereas diatom dominated biofilms represented a net CO_2_ sink.

## Introduction

Microbial mats on intertidal sediments are complex and highly organised ecosystems where opposed oxygen and sulphide gradients favour the development of vertically stratified, multicoloured and cohesive layers of different functional groups of microorganisms that display various forms of respiration, from the surface to the depth. The decreasing redox potential, with increasing depth, influences distribution of varied metabolics and respiratory pathways that involve different terminal electron acceptors and display decreasing apparent free energy yields [1].

For example, phototrophic purple sulphur bacteria are generally found in anoxic layers where light can penetrate. Their main metabolism is anoxygenic photosynthesis (without O_2_ release), which uses reduced sulphur compounds as electron donors but they can also be chemolithotrophs, in dark environments, by using sulphide or thiosulphate as electron donor and oxygen as the terminal electron acceptor [2]. In muddy environments, they commonly proliferate in a layer of about 1-2 mm depth that matches their needs both in terms of light and sulphide availability.

However, it has been observed that mass bloom of phototrophic bacteria, forming large coloured mats, can occur at the sediment surface in the intertidal zone [3]. On the Orkney islands [4] and Roscoff Aber Bay [5], these blooms typically occur on sandy sediments locally enriched in organic matter derived from the decomposition of macroalgal deposits. Purple sulphur bacteria develop at the air/water sediment interface in these systems and are exposed to oxygen, forming large bright pink mats visible to the naked eye.

These mats have been observed widely but the current knowledge about their ecology, ecophysiology and their role in ecosystem functioning is intriguingly far less documented than cyanobacterial and/or diatoms biofilms [5]. This lack of knowledge should be addressed as most of anoxygenic phototrophic bacteria have competitive advantages, such as a high metabolic versatility, which explains their wide distribution and their massive development in various ecological niches [3]. Their role in important ecosystem functions, such as carbon budgets, is currently poorly known.

To better understand the proliferation of purple sulphur bacteria and their influence on important ecosystem functions and services, we compared three different types of mats representative of our study site. These mats were selected on the basis of visibly contrasting densities of purple sulphur bacteria at their surface (i.e. bare sediment, medium and high density). Their actual diversity was examined using a set of biochemical markers (fatty acids, pigments) and their functioning evaluated both in terms of *in situ* total carbon fluxes and sediment surface adhesion (a proxy for surface mat cohesion).

## Materials and Methods

### Study site and sampling

**Figure 1:**
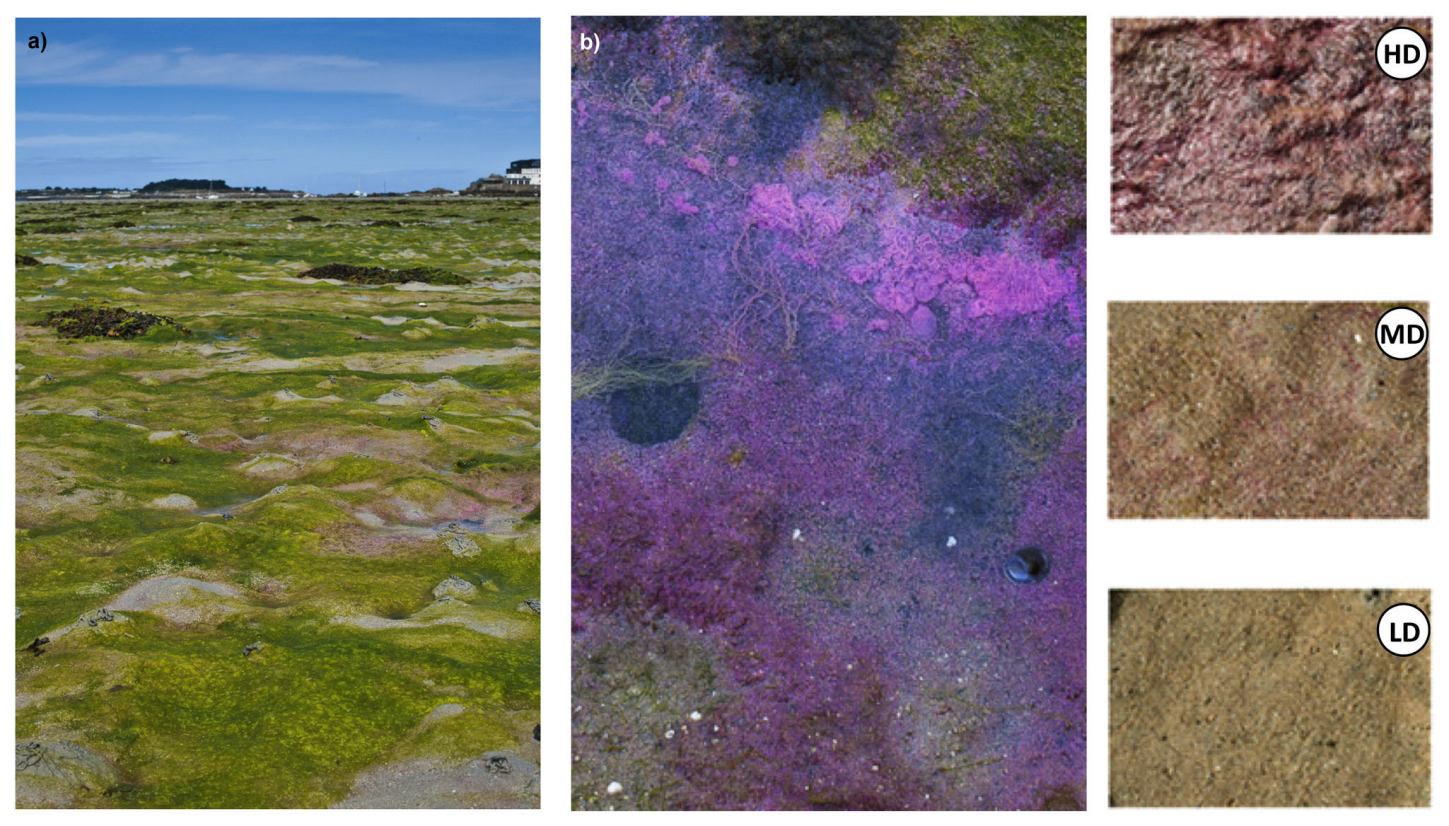
Picture of the Roscoff Aber bay affected by green macroalgae deposits (a) with details of a purple sulphur bacterial mat on top of magroalgal deposits (b), and details of three different types of mats selected for the study (HD: high density, MD: medium density, and LD: low density of purple sulphur bacteria).

No specific permissions were required for the completion of this study as the field measurements did not involve endangered or protected species nor were conducted in a specified protected area. Sediment samples were collected in August 2010 and July 2011 using sterile 10 or 20 ml polycarbonate syringes in the Roscoff Aber Bay (Brittany, France). The bay, is about 1 km long and 2 km wide, entirely situated above mid-tide level, it contains a range of types of intertidal sediments, and has been extensively studied in the past for various aspects of its ecology such as annual carbon budgets [6], biogeochemistry [7] and microbial ecology [8]. The Roscoff Aber bay is also annually affected by the proliferation of green ephemeral macroalgae. Such deposits can account for 10 to 20 % of the entire surface of the bay and affect both the trophic ecology [9] and C fluxes of the ecosystem [6]. Three different types of mats were selected for the study. The presence or absence of visible patches of purple bacteria was deliberately used as selection criteria (Figure 1). All of the selected mats were established on fine-sand sediments (mean particle size: 215 ± 43 μm, [7]). Low-density mats (LD) corresponded to bare sediments whose surface did not display any visible patches of purple sulphur bacteria (Figure 1); medium density (MD) mats corresponded to bare sediments with several patches of purple sulphur bacteria at the surface (about 50% surface covered); and high density (HD) mats corresponded to similar sediments with green macroalgae deposits (about 0.5 cm thick) covered by a thick layer of purple sulphur bacteria (100% of surface covered). LD and MD mats were characterized by oxic layers of about 1-2 cm and <1 cm respectively. Oxic layers were absent or very thin (< 1mm thick) in HD mats (rough estimates).

### Extracellular polymeric substances (EPS)

Triplicate sediment cores were sampled in every microbial mat (2 cm^2^, 3 cm depth, August 2010). Samples were immediately frozen in the field using liquid nitrogen and stored at -80 °C until further analysis. After thawing, sediment cores were mixed with 2 ml of distilled water and continuously rotated for 1.5 h (Horizontal mixer, RM5-30V, Ingenieurbüro CAT), allowing the extraction of colloidal EPS (soluble fraction). The supernatant was removed and sediment was mixed again with 2ml of distilled water and 500 mg of Dowex Marathon C exchange resin to extract bound EPS (attached to sediment particles). Both fractions were then analysed by colorimetry for carbohydrates and proteins. Carbohydrates and proteins were analysed according to the Dubois method [10] and a modified Lowry method [11] respectively.

### Pigment composition

The top 2 mm of the sediment were frozen in the field with liquid nitrogen using the contact core technique [12]. The samples were freeze-dried in the laboratory and kept at -80 °C until further analysis (15 replicates per each type of mats, 2 cm^2^, 2 mm depth, August 2010). Pigments were extracted from a sub-sample of approximately 0.2 g of freeze-dried sediment with 2 ml of 95 % cold buffered methanol (2 % ammonium acetate) for 15 min at ‒20 °C, in the dark. Samples were sonicated for 30 s at the beginning of the extraction period. Extracts were filtered with Whatman membrane filters (0.2 µm) immediately before High Performance Liquid Chromatography (HPLC) analysis. Pigment extracts were analyzed using a Shimadzu HPLC comprised of a solvent delivery module (LC-10ADVP) with system controller (SCL-10AVP), a photodiode array (SPD-M10AVP) and a fluorescence detector (RF-10AXL). Chromatographic separation was carried out using a C18 column for reverse phase chromatography (Supelcosil, 25 cm long, 4.6 mm in diameter, and 5 µm particles). The solvents used were 0.5 M ammonium acetate in methanol and water (85:15, v:v), acetonitrile and water (90:10, v:v), and 100 % ethyl acetate. The solvent gradient followed Kraay et al. [13] adapted by Brotas and Plante-Cuny [14] with a flow rate of 0.6 ml min^-1^ and an injection volume of 100 µl. Identification and calibration of the HPLC peaks was confirmed with chlorophyll *a* (chl*a*), chlorophyll b (chl*b*) and b-carotene standards from Sigma and chlorophyll *c* (chl*c*), fucoxanthin (fuco), diadinoxanthin (DD), diatoxanthin (DT), lutein (lut), zeaxanthin (zea), and pheophytin a standards from DHI. Bacteriochlorophyll *a* (BChl*a*) was identified and quantified using a standard from Frontier Scientific. Pigments were identified by the absorption spectra and retention times and the concentrations calculated from the signals in the photodiode array (chlorophylls and carotenoids) or fluorescence detector (pheophorbides and pheophythins).

### Fatty acid composition

Fatty Acids (FAs) were extracted from 3 cm depth freeze-dried sediment cores (triplicates for each type of biofilm, August 2010) following the method of Bligh and Dyer [15] slightly modified by Meziane et al. [16]. In addition, several sediment cores were sliced with a thin blade at 0.5 cm intervals and FAs were extracted from each layer (triplicates per each type of mat, 2 cm^2^ inner diameter cut off syringe, in July 2011). Lipids were extracted by sonication (35 kHz, 20 min) using a chloroform/methanol/water cocktail (2:1:1, v:v:v). An internal standard (tricosanoic acid: 23:0, 10 µg) was added to each sample before extraction. Lipids were concentrated under a constant N_2_ flow, and the residue saponified (90 min, 90 °C) with NaOH:MeOH (1:2, v:v). Saponification and methylation procedures were applied to transform the total lipids into FA-methyl esters [17] so that the FAs can be separated and quantified by gas chromatography (GC, Varian CP-3800 equipped with flame ionization detector). Most FAs were identified by comparing their retention times with those of known standards (Supelco™ 37, PUFA-1 Marine Source, and Bacterial Mix; Supelco Inc., Bellefonte, PA, USA). Unidentified FAs were further identified by GC coupled to a mass spectrometer (GC-MS, Varian GC450-220MS). For both devices, FAs separation was performed using a Supelco OMEGAWAX 320 column (30 m x 0.32 mm i.d., 0.25 µm film thickness) with H_2_ as carrier gas. After injection of 1 µl of sample at 60 °C, the temperature was raised to 150 °C at 40 °C min^-1^, then to 240 °C (held 14 min) at 3 °C min^-1^. Each FA was given as a percentage of total FAs.

### Sediment stability

Sediment stability was assessed in August 2010 through the proxy of surface adhesion (i.e. stickiness) using Magnetic Particle Induction (MagPI), a device recently developed by Larson et al. [18]. Briefly, a given amount of ferrous and stained ferromagnetic particles (diameter > 425 µm) was spread onto the sediment surface in a single layer. Then, the magnetic force needed to detach the particles from the substratum was measured, using an electromagnet set at a constant distance from the surface (3 mm). The voltage supplied to the magnet, was controlled by a precision power supply (HY3005 DC Power Supply, Mastech) and was increased from 0 V by increments of 0.1 V. When all particles detached from sediment the final voltage was recorded and used to calculate the corresponding magnetic force (mTesla). A calibration curve was previously established with a gaussmeter (410-HCAT, LakeShore) by measuring the magnetic force displayed at each incremental increase of the voltage. Five replicates were sampled in every microbial mat, using a cut-off syringe and without disturbing the sediment surface. The cores were maintained vertically, transferred to laboratory and immediately analysed. MagPI measurements have been previously correlated with cohesive strength meter (CSM) measurements which give the sheer stress needed to erode the sediment (Newton.m^-2^, [19]). 

### Carbon fluxes

Carbon fluxes were measured *in situ*, in August 2010, during low tide (air-sediment interface), with a benthic chamber [20]. The chamber covered a 0.07 m^2^ sediment area and consisted of a stainless-steel ring pushed into the emersed sediment to about 10 cm depth. It was hermetically sealed by either a transparent or dark Perspex® dome. The chamber was then connected to a closed gas circuit for about 15-30 min in order to record changes in CO_2_ partial pressure (data logger LiCor Li-1400, with a 30s logging frequency). CO_2_ was monitored with an infrared gas analyzer (LiCor Li 800) in ppm. From recorded data of either light or dark incubations, CO_2_ partial pressure was regressed against time. Slopes of the corresponding regression curves were used to calculate respectively the net primary production and respiration. Results were then expressed in carbon units (mgC m^-2^ h^-1^). Incident photosynthetically active radiation (400-700 nm, PAR in µmol quanta m^-2^ s^-1^) was also measured with a LiCor quantum sensor.

### Statistical analyses

All statistical analyses and graphs were performed using R© version 2.14.0. FAs and pigment compositions of the sampled mats were compared separately using multivariate analyses that allowed both sampling sites and metabolites scores to be displayed in the same plot (i.e. non metric multidimensional scaling – nMDS). The relative amounts of pigments and FAs were transformed using a Hellinger transformation. Differences in FA or pigment composition between biofilms were tested statistically by one-way permutational multivariate analysis of variance (permanova) after proper verification for multivariate homogeneity of group dispersions (tested with a permutation-based test). Differences between mats of individual FA, pigments as well as EPS, CO_2_ fluxes, sediment stability were tested using Kruskal-Wallis test (one way non parametric ANOVA) followed by pairwise Wilcoxon-Mann-Whitney (WMW) tests adjusted by the Benjamini Hochberg (BH) method (non parametric post-hoc test).

## Results

### Quantification of pigments and FA markers

**Figure 2:**
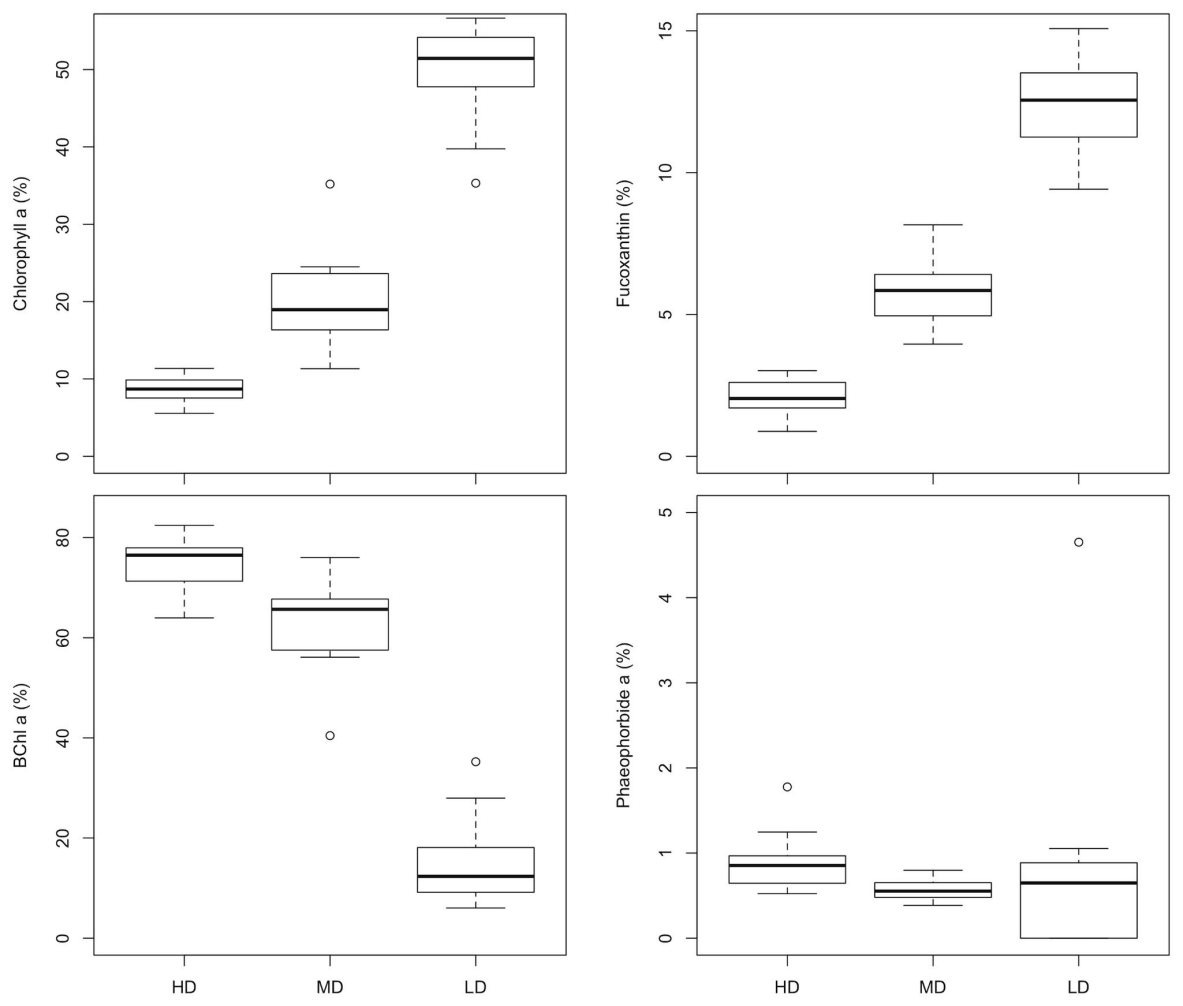
Proportion of Chlorophyll a, Fucoxanthin, Bacteriochlorophyll a (BChl a) and Pheophorbide a (in %) to the total pigment pool in HD, MD and LD microbial mats.

As expected from visual inspection, bacteriochlorophyll *a* (BChla) increased gradually from LD (0.8 ± 0.7 µg.g^-1^) to HD mats (300 ± 141 µg.g^-1^). Identically, chlorophyll *a* (Chla) concentrations were higher in HD biofilms. In addition, HD mats were also characterised by high amounts of degraded chlorophyll (i.e pheophorbide *a*, 3.4 ± 1.8 µg.g^-1^), in comparison to MD and LD mats (0.15 ± 0.08 and 0.05 ± 0.09 µg.g^-1^). These pigment concentrations (as well as all the detected pigments) were significantly different between the mats and significantly higher in HD mats (Kruskal-Wallis tests, p < 0.001, followed by pairwise WMW tests). However, in term of pigment contribution to the total pool (Figure 2), Chla and fucoxanthin represented only a small percentage of the total pigments pool in HD mats (8 ± 2 % and 2 ± 1 % respectively) in comparison to LD mats (50 ± 6 % and 13 ± 2 % respectively). Conversely, BChla contribution to the total pigment pool was higher in HD mats (75 ± 5 %) than in LD mats (15 ± 8 %). 

**Figure 3:**
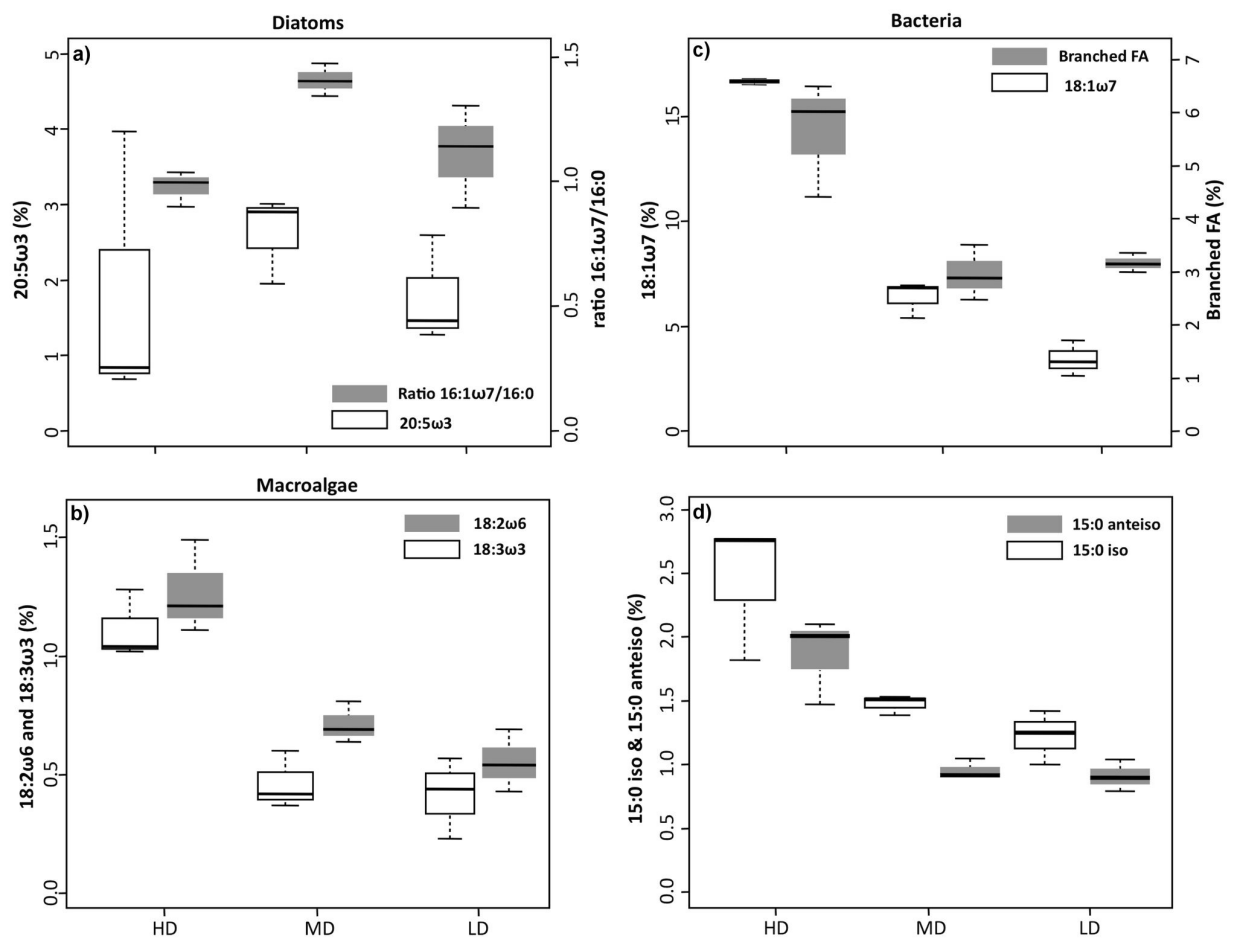
Proportion of several fatty acids (in %) to the total FA pool in HD, MD and LD microbial mats. a) 16:1ω7/16:0 ratio and 20:5ω3, b) 18:2ω6 and 18:3ω3, c) sum of all the branched FAs (i.e. iso and anteiso fatty acids) and 18:1ω7 d) 15:0iso and 15:0anteiso. These fatty acids are respectively indicators or biomarkers of diatoms (a), macroalgae (b), and bacteria (c and d).

Eicosapentaenoic fatty acid (20:5ω3) levels and the ratio of palmetic to palmitoleic acid (16:0/16:1ω7) were used as markers for benthic diatoms [21]. While Chla contribution decreased from LD mats to HD, these two markers did not follow the same pattern (Figure 3). Each marker was more abundant in MD than in LD mats but no significant difference were found between HD and LD mats (WMW, p > 0.05). In addition, HD mats contained the highest amounts of branched FAs (i.e. sum of all iso and anteiso FAs), which are typically used as markers of bacteria [22,23], and vaccenic acid (18:1ω7), which is typically used as marker of purple bacteria [24]. The proportion of each marker was significantly different between the mats considered (Kruskal-Wallis tests, p < 0.001, followed by pairwise WMW tests). The 15:0 iso and 15:0 anteiso branched FA were significantly more abundant in HD and MD cores than LD cores (Kruskal-Wallis tests, p < 0.001, followed by pairwise WMW tests). Finally, the proportion of linolenic fatty acids (18:3ω3 and 18:2ω6) was significantly different between the mats considered; with HD biofilms displaying the highest percentages (Kruskal-Wallis tests, p < 0.001, followed by pairwise WMW tests). No significant difference was found between LD and MD biofilms in term of 18:3ω3 content (WMW, p > 0.05).

### Distribution of pigment and FAs in mats

**Figure 4:**
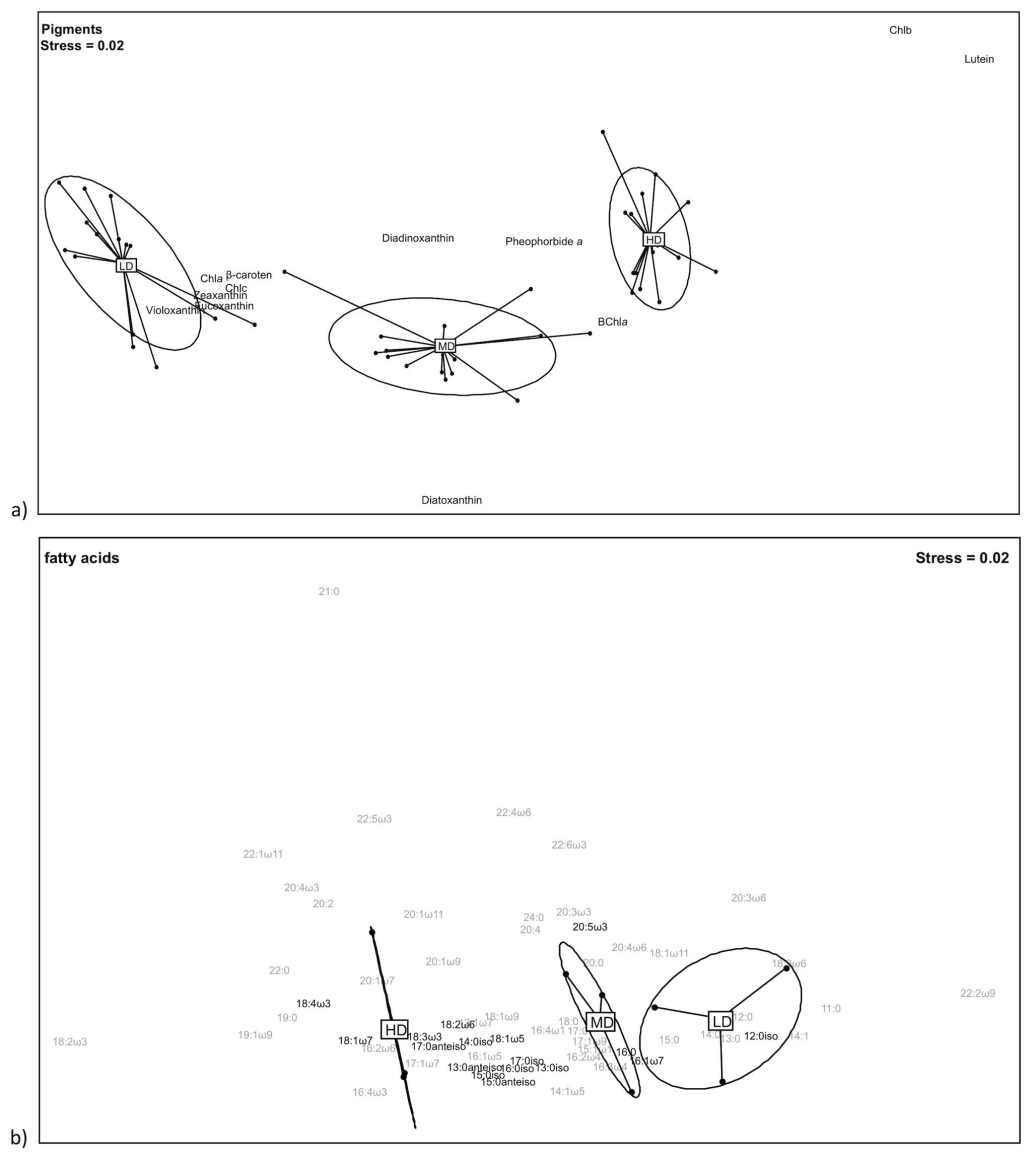
Non-metric multidimensional scalings (nMDS) calculated with pigments (a) and fatty acids (b) proportions (in %). The stress value indicates the quality of the 2D representation. The ellipses are intended to group replicated of the same type of microbial mat together. Minor fatty acids (b) were coloured in gray.

The non-metric multidimensional scaling (nMDS, stress = 0.04, Figure 4a). The nMDS showed that the pigment composition in studied mats was significantly different (permanova, p < 0.01), MD displayed intermediate pigment composition between LD and HD mats. HD mats were characterised mainly by degraded chlorophyll (pheophorbide *a*), BChla and Chlorophyll b (Chlb). LD mats were clearly separated by fucoxanthin, Chla, chlorophyll *c* (Chlc). MD mats were distinguished by high diatoxanthin and diadinoxanthin contents.

A nMDS also showed that the FA composition in mats was significantly different (Figure 4b, stress = 0.02), LD being opposed to HD, and MD biofilms being intermediate between LD and HD. HD mats were clearly characterised by most of the bacterial markers (branched FAs), macroalgal markers (18:3ω3, 18:3ω6) and the vaccenic fatty acid (18:1ω7). LD mats were distinguished by benthic diatoms markers (20:5ω3; 16:0, 16:1ω7). Taking into account the whole dataset, HD, MD and LD FAs compositions were significantly different (permanova, p < 0.001).

### Depth distribution of major bacterial FAs markers

**Figure 5:**
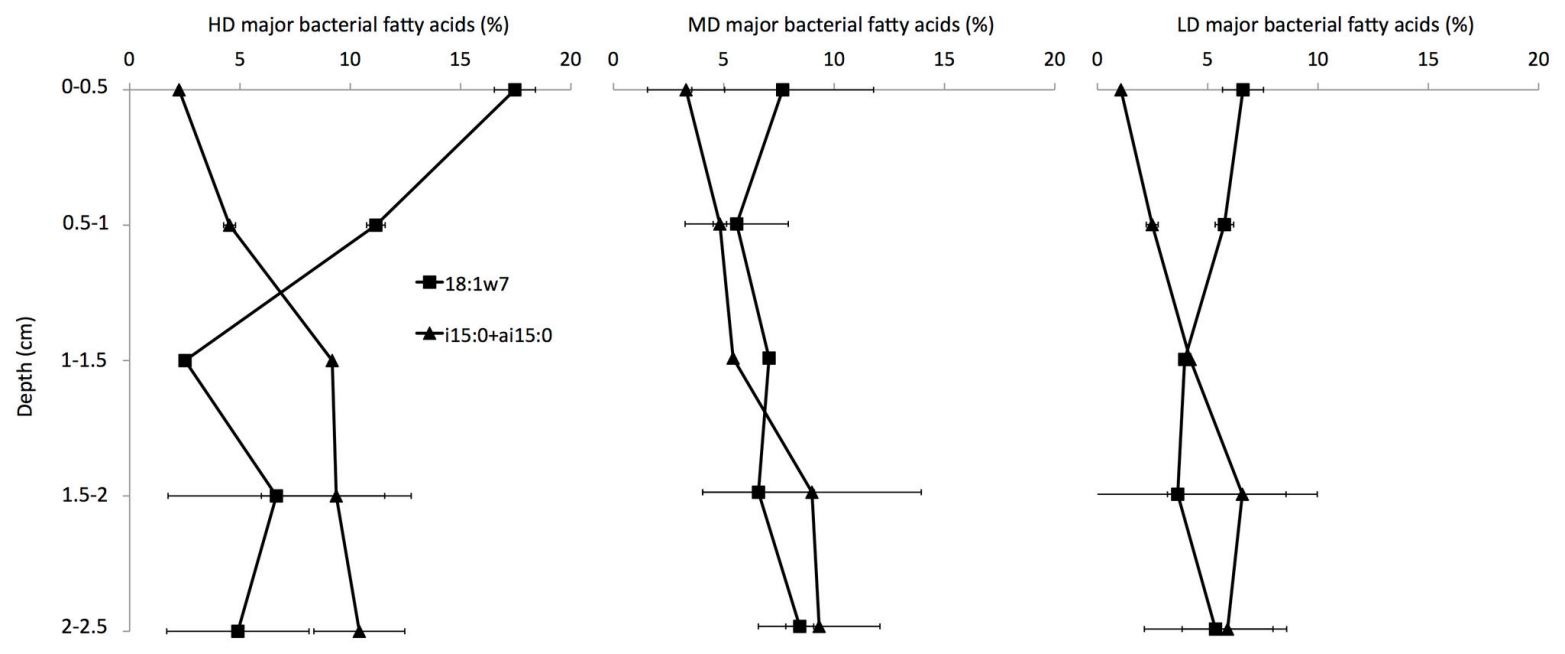
Depth distribution of major bacterial FAs in HD, MD and LD mats.

A steep gradient of vaccenic acid (18:1ω7) was observed from surface (up to 18 % of total FAs) to 1-1.5 cm depth (about 2.5 %) in HD cores whereas its distribution was relatively homogenous along LD and MD cores (Figure 5). Simultaneously, the contribution of 15:0 iso and 15:0 anteiso branched FAs increased from the surface (2.2 %) to 1 cm depth (9.2 %).

### Exopolymers (EPS) and sediment surface adhesion

**Figure 6:**
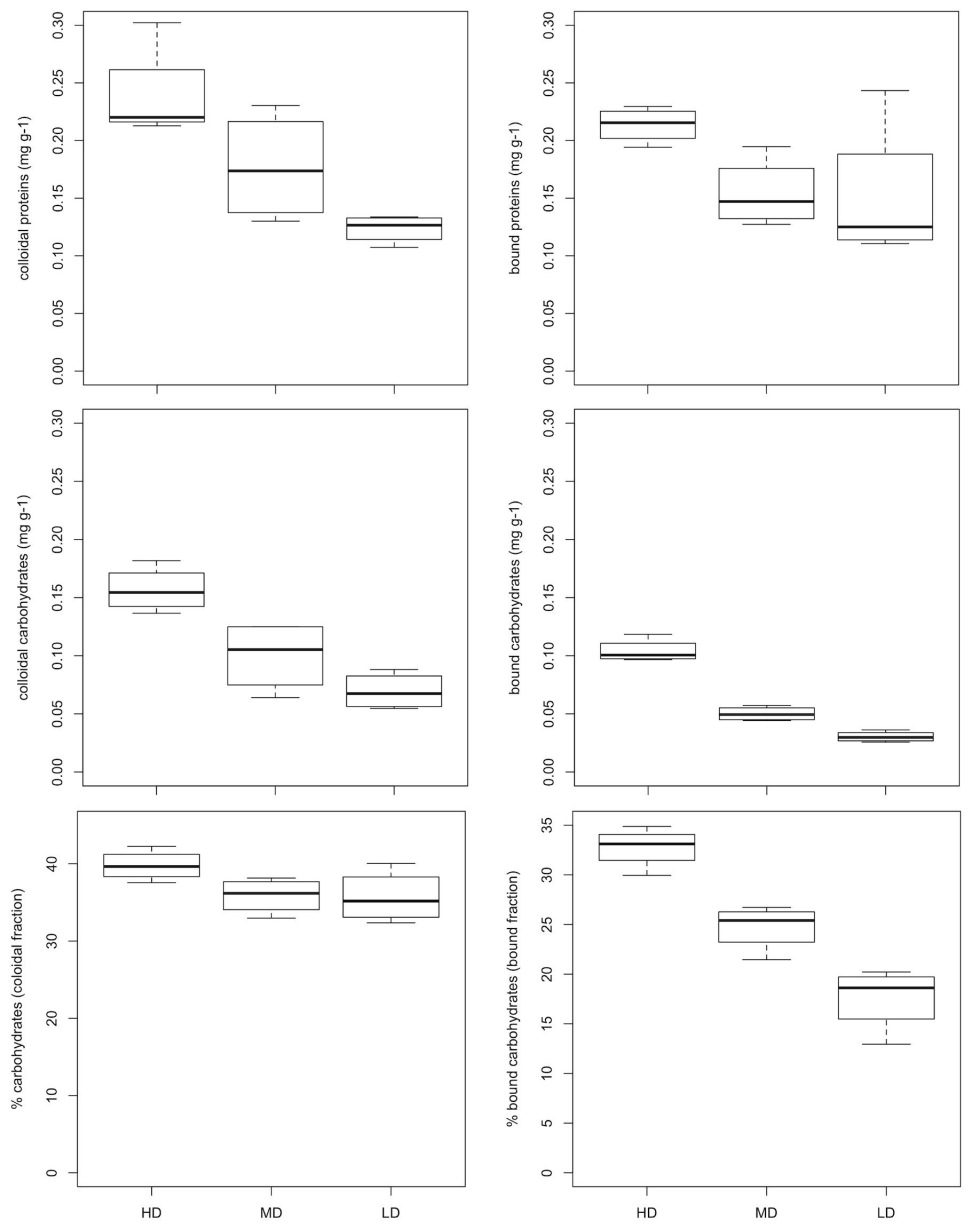
Extracellular polymeric substances (EPS) in HD; MD and LD mats. Colloidal and bound extracellular proteins (top), colloidal and bound extracellular carbohydrates (middle), and proportion of carbohydrates (in %) to the toal EPS pool in the colloidal and bound fractions (bottom).

**Figure 7:**
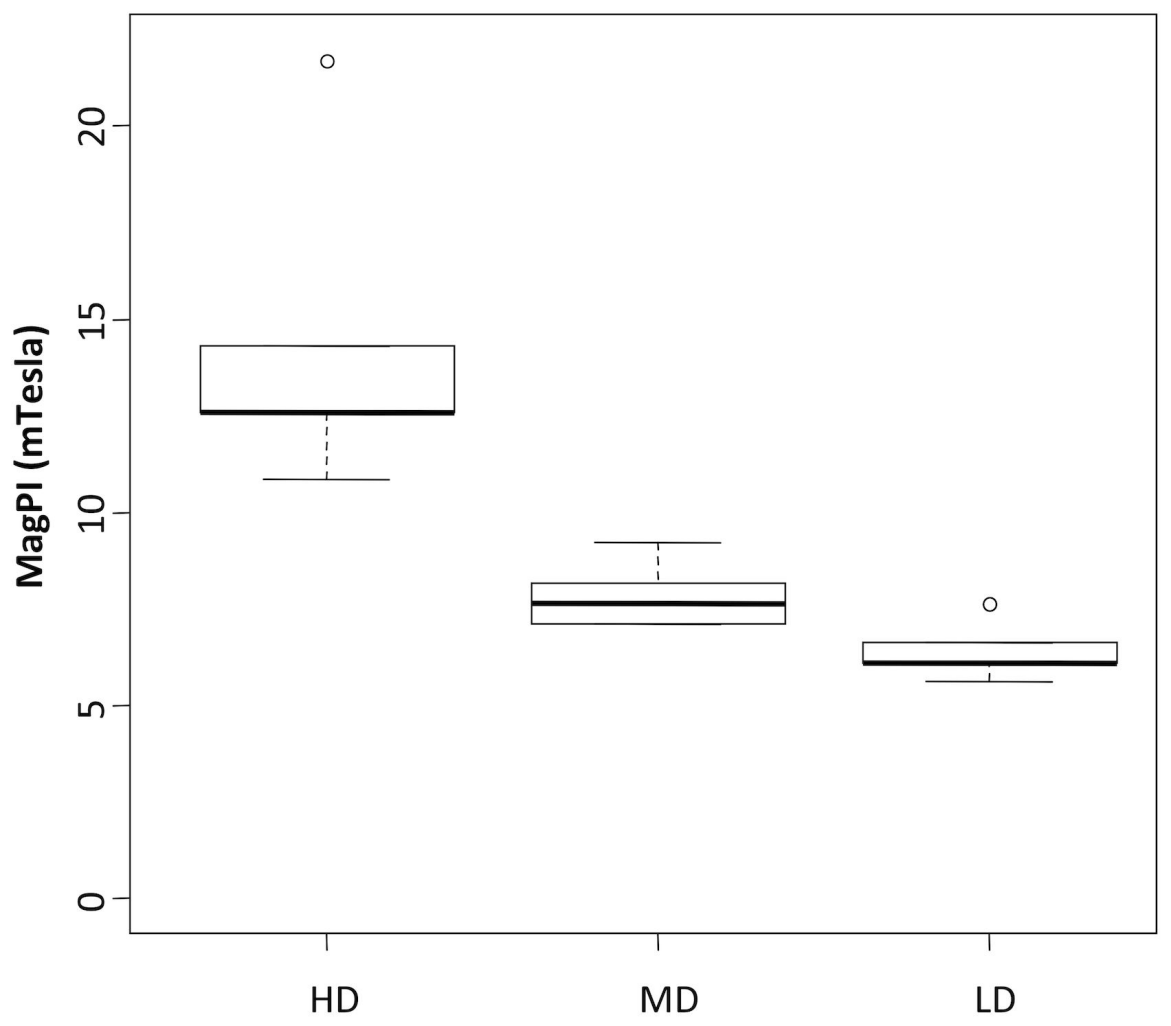
Magnetic Particle induction (MagPI) of HD, MD and LD biofilms. The measure is a proxy for surface mat cohesion.

For each EPS fraction (i.e. colloidal and bounded) (Kruskal-Wallis tests, p < 0.05, followed by pairwise WMW tests), carbohydrates and proteins concentrations were higher in HD mats than in LD mats (Figure 6). In all samples, carbohydrate contribution to either total colloidal or bound EPS was always lower than 50 %. For the colloidal fraction, no significant differences were found between mats, whereas bound carbohydrates were significantly higher in HD biofilms than in MD and LD mats (Figure 6, Kruskal-Wallis tests, p < 0.05, followed by pairwise WMW tests). Surface adhesion followed the same pattern as EPS contents being significantly higher in HD biofilms than MD and LD mats (Figure 7, Kruskal-Wallis tests, p < 0.05, followed by pairwise WMW tests). 

### Carbon fluxes

**Figure 8:**
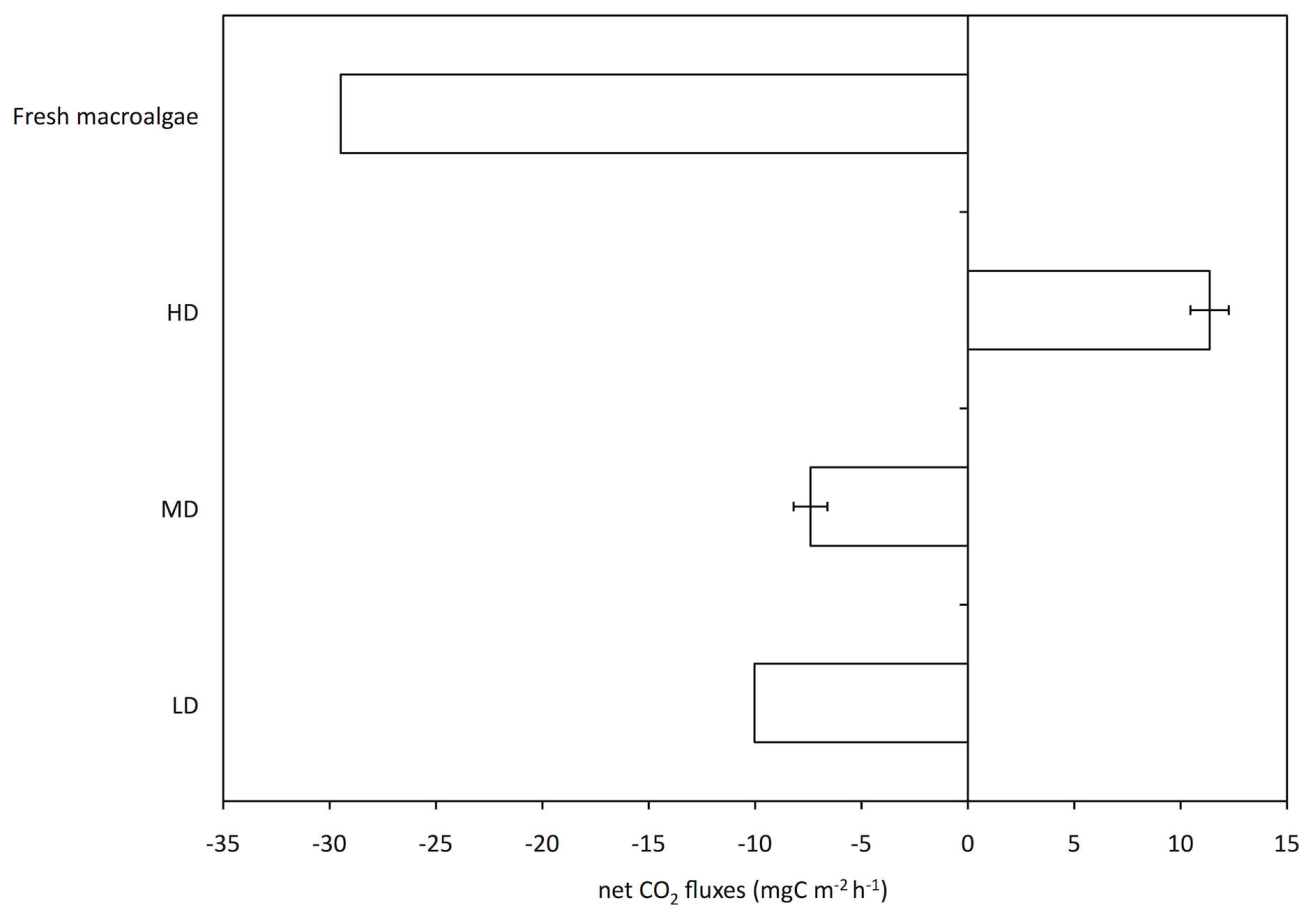
Net CO_2_ fluxes at the air-sediment interface in HD, MD and LD mats as well as in fresh macroalgal deposits. These fluxes were measured using a benthic chamber. Negative fluxes indicate a net fixation of inorganic carbon (i.e. CO_2_) whereas positive fluxes indicate a CO_2_ degassing.

In opposition to MD and LD mats (CO_2_ sinks), HD mats displayed positive net CO_2_ fluxes (CO_2_ source). HD fluxes were significantly higher than MD (WMW, p < 0.05) and LD mats fluxes. Net CO_2_ fluxes recorded on fresh macroalgae deposits (i.e. macroalgal layer similar to that of HD mats but without purple sulphur bacteria at their surface) were highly negative (Figure 8). 

## Discussion

### Microbial composition of intertidal mats

Fatty acids and pigment compositions of the Roscoff Aber bay mats displayed striking differences as shown by the permutational analyses of variance. Each type of biochemical marker could be considered as a “chemical fingerprint” of the mat, which carry taxonomic information about the taxonomy of all living organisms (fatty acids) or only phototrophs (pigments). These fingerprints are, to some extent, equivalent to “genetic fingerprint” (such as DGGE, TTGE) as they provide similar information but with different taxonomic resolutions. Based on this fingerprinting approach, multivariate analyses clearly showed that the opposition between HD and LD mat composition mirrored the functional variation between anoxygenic and oxygenic photosynthesis. 

HD mats were characterized by the almost exclusive presence of purple bacteria as shown by the relatively high contributions of vaccenic acid to the FA pool (i.e. 18:1ω7 represented 16.7 ± 0.2 % in HD biofilms). Although present in most of bacteria, this FA is found in high proportions in anoxygenic bacteria [24] and several studies suggest it is diagnostic for sulphur-oxidizing bacteria in H_2_S-rich marine sediments (e.g. 25,26). This is consistent with a preliminary study [5] which showed that HD mats were mainly composed of bacteria belonging to the *Chromatiaceae* family (based on 16S rRNA and *pufM* cDNA libraries, unpublished results). Mass blooms similar to those that develop annually in the Roscoff Aber Bay were observed in summer in the intertidal zone of sandy beaches [27–29] where bacterial abundances reached up to 10^7^ cells.cm^-3^ [28]. Although they contained the highest Chla concentrations, HD biofilms were also characterised by the relative poor contribution of diatom markers, such as the 16:1ω7/16:0 ratio [21], to the FA pool (0.97 ± 0.07 %). Contrary to LD and MD mats, most of the Chla in HD cores originated from deposited macroalgae (as shown by the low 16:1ω7/16:0 ratio and the relative absence of 20:5ω3 in HD biofilms). Consistent with our fled observations, the high concentrations of pheophorbide *a* in these cores indicated that despite a significant part of the deposited macroalgae being degraded, some fresh macroalgae remained still active under the purple bacterial layer. The presence in HD mats of linolenic fatty acids (18:3ω3 and 18:2ω6), which are synthesised in high proportion in green macroalgae [21] and FAs 20:4ω3 or 22:5ω3 and 22:4ω6 (Figure 4) intermediate in the biosynthesis of 20:5ω3 or 22:6ω3 [30] in marine algae are in line with this hypothesis.

MD and HD cores were also characterised by their high content of 15:0iso and 15:0anteiso branched FAs below 1 cm depth. These compounds are abundantly found in sulfate-reducing bacteria (SRB) such as *Desulfovibrio* sp. [31]. In these cores, purple sulphur bacteria (represented by 18:1ω7) were predominantly found at the sediment surface, suggesting that they used reduced sulphur compounds from sulphate reduction.

Surprisingly, although LD cores did not display any purple patches at their surface, small amounts of BChla (0.99 ± 0.76 mg.m^-2^) and vaccenic acid (3.43 ± 0.85 %) were detected (one sample wilcoxon signed rank tests, p < 0.05). In these mats, BChla represented on average 14 % of the total pigment pool. This finding is consistent with a previous study that showed the presence of purple bacteria at the surface of similar sediments by spectral reflectance measurements [5]. Thus, altogether three different markers: 18:1ω7 and BChla (this study), and remote sensing measurements [5] are consistent and indicate that the selection criteria of our mats (based on mat colour) was accurate and reflects the biomass of purple sulphur bacteria. This demonstrates that purple sulphur bacteria are able to thrive on relatively well-oxygenated sediments suggesting that their role and functions in intertidal sediments is probably underestimated.

### Biostabilisation of sandy sediments by purple bacteria

Sediment stability is an important feature in ecosystems subjected to fluctuating hydro-sedimentary regimes such as present in intertidal areas. In this context, microbial exopolymeric secretion is recognized as a major stabilising factor in the tidal zone [32]. EPS are ubiquitous component of marine ecosystems primarily composed of carbohydrates, proteins and lower amounts of other components [33]. In our study, we observed that both bound and colloidal proteins and carbohydrates were significantly more abundant in HD biofilms than MD or LD biofilms. These molecules, which are usually mostly produced by diatoms in the intertidal area [32], compose a highly hydrated matrix, which physically binds sediment grains together. As a result, the cohesiveness of the sediment is generally increased (e.g. 19,33). We demonstrated here that the surface adhesion (a proxy of sediment stability as shown by [19]) was indeed higher in HD mats than MD and LD mats, following the same pattern as EPS, carbohydrates, and proteins as well as BChla. Although we cannot exclude entirely that green macroalgae and heterotrophic bacteria also contribute to EPS formation, we assume that purple sulphur bacteria are the main producers in HD mats. Altogether these data suggest that sediment particle adhesion is enhanced when purple sulphur bacteria develop at the sediment surface. Sediment binding by purple sulphur bacteria has been hypothesised before [28] and observed in laboratory flume experiments [34]. Here our field experiments demonstrated that sediment erosion is expected to decrease when exopolymers from purple phototrophic mats are present.

### Role of purple bacterial mats in net CO_2_ fluxes

In the present study, net CO_2_ flux measurements represented the balance between autotrophic (CO_2_ fixation) and heterotrophic processes (CO_2_ production), for the whole benthic community, during low tide. Net CO_2_ flux was significantly more negative on fresh macroalgal deposits than on any of the studied mats. This is consistent with a previous study [6] that showed that macroalgal deposit consistently increased the *in situ* gross primary production and that, at an hourly scale, the impact of opportunistic macroalgae deposits on benthic metabolism was very significant. LD and MD biofilms also displayed negative net CO_2_ fluxes but considerably lower in magnitude than on fresh green macroalgae. Due to the absence of replicates it was not possible to compare these fluxes statistically. However they were consistent with previous measurements in the Roscoff Aber Bay [6,7].

Contrary to the other mats, HD mats displayed positive net CO_2_ fluxes, which indicated the predominance of CO_2_ producing and/or heterotrophic processes. According to previous measurements, these mats were dominated by metabolically versatile species of the Chromatiaceae family. These species are mainly photolithoautotrophic under anoxic conditions using different photosynthetic electron donors such as thiosulphate, sulphide and elemental sulphur, and can also grow photoorganoheterotrophically in the absence of reduced sulphur [35]. Some species, such as *Thiocapsa roseopersicina*, can also grow as chemolithoautotrophs on reduced sulphur compounds [36]. In addition, a large number of organic carbon sources (including acetate and pyruvate) can be used by the versatile species of the Chromatiaceae [35]. Despite a high Bchla production, purple sulphur bacterial mats were a CO_2_ source rather than a CO_2_ sink. This is consistent with Al-Najjar et al. [37] who demonstrated that light energy conservation is generally low (< 1%) at high irradiance in different photosynthetic microbial mats, and that most of the absorbed light energy is wasted as heat when light is available in excess. According to these authors, we suggest that, at low tide, photosynthetic efficiency was probably hampered in our mats by the high incident irradiance and that OM inputs from macroalgal deposits favour the growth of purple sulphur bacteria via organo-heterotrophic pathways. We hypothesise that photosynthetic efficiency would increase at high tide due to the attenuation of light within the water column. The massive growth of purple sulphur bacteria in tidal systems is thus potentially explained by the capacity of the bacterial mats to shift between phototrophic (high tide) versus heterotrophic (low tide) metabolisms in response to the tidal cycle.
